# DNA metabarcoding reveals that coyotes in New York City consume wide variety of native prey species and human food

**DOI:** 10.7717/peerj.13788

**Published:** 2022-09-21

**Authors:** Carol S. Henger, Emily Hargous, Christopher M. Nagy, Mark Weckel, Claudia Wultsch, Konstantinos Krampis, Neil Duncan, Linda Gormezano, Jason Munshi-South

**Affiliations:** 1 Louis Calder Biological Field Station, Fordham University, Armonk, New York, United States; 2 Mianus River Gorge, Bedford, New York, United States; 3 American Museum of Natural History, New York, New York, United States; 4 Bioinformatics and Computational Genomics Laboratory, City University of New York, Hunter College, New York, New York, United States; 5 Department of Biological Sciences, City University of New York, Hunter College, New York, New York, United States; 6 Institute of Computational Biomedicine, Weill Medical College of Cornell University, New York, New York, United States

**Keywords:** DNA metabarcoding, Coyotes, Diet, New York City, Urbanization, Noninvasive genetic sampling

## Abstract

Carnivores are currently colonizing cities where they were previously absent. These urban environments are novel ecosystems characterized by habitat degradation and fragmentation, availability of human food, and different prey assemblages than surrounding areas. Coyotes (*Canis latrans*) established a breeding population in New York City (NYC) over the last few decades, but their ecology within NYC is poorly understood. In this study, we used non-invasive scat sampling and DNA metabarcoding to profile vertebrate, invertebrate, and plant dietary items with the goal to compare the diets of urban coyotes to those inhabiting non-urban areas. We found that both urban and non-urban coyotes consumed a variety of plants and animals as well as human food. Raccoons (*Procyon lotor*) were an important food item for coyotes within and outside NYC. In contrast, white-tailed deer (*Odocoileus virginianus*) were mainly eaten by coyotes inhabiting non-urban areas. Domestic chicken (*Gallus gallus*) was the human food item found in most scats from both urban and non-urban coyotes. Domestic cats (*Felis catus*) were consumed by urban coyotes but were detected in only a small proportion of the scats (<5%), which differs markedly from high rates of cat depredation in some other cities. In addition, we compared our genetic metabarcoding analysis to a morphological analysis of the same scat samples. We found that the detection similarity between the two methods was low and it varied depending on the type of diet item.

## Introduction

Urbanization results in the fragmentation of native ecosystems and creates a mosaic of green spaces differing in size and shape and surrounded by human development (Norton, Evans & Warren, 2016). The heterogeneity of the urban environment alters ecological communities and generally decreases native biodiversity (Medley, McDonnell & Pickett, 1995; Natuhara & Imai, 1996; Clergeau, 1998; Luck, 2007). For example, diet and habitat specialists are usually not able to live in urban areas because of spatial and temporal variation of the environment (Clavel, Julliard & Devictor, 2011). In contrast, generalist species thrive in urban environments because they can utilize a variety of different habitats and food resources (Kark et al., 2007). Additionally, many large and mid-sized animals require larger contiguous habitats than are available in urban environments (Saito & Koike, 2013).

Changing urban community composition also affects predator-prey relationships. Urban assemblages of prey species differ from those in non-urban areas in that they contain more human commensals and invasive species, such as brown rats (*Rattus norvegicus*), European starlings (*Sturnus vulgaris*), pigeons (*Columba livia*), American crows (*Corvus brachyrhynchos*), and domestic cats (*Felis catus;* McKinney, 2006). In addition, some mesocarnivores, such as raccoons (*Procyon lotor*) and striped skunks (*Mephitis mephitis*), reach higher population densities in urban areas than non-urban ones (Gehrt, Riley & Cypher, 2010). Larger prey species, such as white-tailed deer (*Odocoileus virginianus*), while quite abundant in suburban and exurban areas, are less prevalent in truly urban landscapes due to a lack of suitable vegetation in the manicured understories within urban green spaces such as recreational parks, golf courses, and cemeteries (Gallo et al., 2017).

The increased impervious surface and smaller size of urban habitats may decrease the diversity of prey species that predators consume (Grimm et al., 2008). For example, a comparison of mammal species diversity among different land use types in Raleigh, N.C. and Washington, D.C. showed that suburban and exurban areas had higher species diversity than urban areas (Parsons et al., 2018). Similarly, mammalian species richness has been shown to decline with an increase in impervious surfaces (Dickman, 1987) and bird species richness to decline with increasing urbanization over time (Strohbach, Hrycyna & Warren, 2014).

Food provided by humans through outside composting (Murray et al., 2016), trash (Oro et al., 2013), bird feeders (Fuller et al., 2008), and pet food (Contesse et al., 2004) are also abundant in all developed areas, including highly urbanized areas like large cities. As a result, some urban wildlife species may alter their diets to include these novel resources. For example, more than half of the diet of urban red foxes (*Vulpes vulpes*) in Zürich consisted of anthropogenic food, such as processed meat, bread, pasta, and cheese (Contesse et al., 2004). American white ibis (*Eudocimus albus*) were also found to eat more anthropogenic food (bread and chips) as the level of urbanization in their habitat increased. (Murray et al., 2018). Predictable food subsidies from humans can cause ecological changes, including higher population densities, changes in foraging behavior, and altered dispersal, migration, and gene flow (Oro et al., 2013). Human food provisioning can also lead to human-wildlife conflict if animals associate humans with food. Human-wildlife conflicts may be of particular concern when predator species are involved. For instance, increases in coyote (*Canis latrans*) attacks on humans was related to coyote habituation to humans through either direct or indirect feeding (Timm et al., 2004; Lukasik & Alexander, 2011). Negative human-wildlife interactions may occur if urban predators consume domestic species such as domestic cats, domestic dogs (*Canis familiaris*), or livestock. For example, Moss, Alldredge & Pauli (2016) found that lethal removal of cougars (*Puma concolor*) was more likely when the cougars were observed near livestock.

Coyotes are predators that live in nearly every city in every state of the contiguous United States, spanning a variety of urban and non-urban areas (Hody & Kays, 2018). Coyotes are opportunistic generalists that will alter their feeding behavior to take advantage of available food items (Andelt et al., 1987). In non-urban areas, coyotes primarily consume cottontail rabbits (*Sylvilagus* spp.), rodents, and white-tailed deer, (MacCracken & Uresh, 1984; Brillhart & Kaufman, 1995; Patterson, Benjamin & Messier, 1998; Bartel & Knowlton, 2005; Smith et al., 2018; Petroelje et al., 2021) and there is some evidence that coyotes may regulate white-tailed deer populations (Chitwood et al., 2015). They also consume vegetation, fruit, birds, insects, and human-associated foods such as dump refuse (Bekoff, 1978). Moose (*Alces alces*) and elk (*Cervus canadensis*), and caribou (*Rangifer tarandus*) also make up a large part of the coyote diet in areas where those species co-occur (Sivy et al., 2018; Gifford, Gese & Parmenter, 2019; Balluffi-Fry, Nowell & Humphries, 2020; Shi et al., 2021). Across a suite of cities, researchers have found that the coyote diet is dominated by rodents and rabbits (*e.g.*, Chicago: Morey, Gese & Gehrt, 2007; Los Angeles: Fedriani, Fuller & Sauvajot, 2001; Tuscon: McClure, Smith & Shaw, 1995), but also includes common urban mammals such as squirrels (*Sciurus* spp.) (Seattle: Quinn, 1997) raccoons (Cleveland: Cepek, 2004), and birds (Morey, Gese & Gehrt, 2007). Urban coyotes will consume deer where available (Los Angeles: Shargo, 1988; Fedriani, Fuller & Sauvajot, 2001; Morey, Gese & Gehrt, 2007), as well as vegetation, fruits, and anthropogenic food. (San Diego: MacCracken, 1982; Quinn, 1997). Generally, coyotes consume more anthropogenically-sourced food in more urbanized areas (McClure, Smith & Shaw, 1995; Fedriani, Fuller & Sauvajot, 2001; Morey, Gese & Gehrt, 2007; Newsome et al., 2015; Larson et al., 2020), including items such as birdseed, dog kibble, domesticated fruit, and bread (MacCracken, 1982; McClure, Smith & Shaw, 1995; Murray et al., 2015). Domestic dogs and cats are sometimes consumed by urban coyotes (Gehrt, Riley & Cypher, 2010). Cats are more prevalent in the coyote diet than dogs, but cat consumption varies widely by geographic region.

The recent founding of a coyote population in NYC provides an excellent opportunity to examine the ecological niche of a mid-sized predator in a highly urbanized system. Coyotes were first documented in NYC in 1994 (Toomey et al., 2012) and by 2016 evidence of breeding groups was recorded (Nagy et al., 2016, 2017). Containing 8.8 million people, NYC is the most densely populated city in the United States (United States Census Bureau, 2020). The dense human population may increase the amount of anthropogenic food available to the coyotes. However, NYC also contains green spaces covering over 30,000 acres of land (NYC Parks Dept, 2022) that can provide naturalistic food items.

Using morphological analysis of scat samples, Duncan et al. (2020) previously found that the percent occurrence (number of scats that contained the diet item/occurrence of all diet items) of diet items in the NYC coyote diet consisted of approximately 36% mammals, 15% birds, 33% plants, 9% invertebrates, and 7% anthropogenic items. The main prey species detected were rodents, deer, rabbits, and racoons (Duncan et al., 2020). Birds were also among the top prey items, but they could only be identified to the class Aves. The morphological diet analysis of prey remains (*e.g.*, bones, teeth) produced much information about mammalian prey items, but was limited in the detection and identification of plants and human food items. In this study, we analyzed the diet of coyotes in the New York Metropolitan area using DNA metabarcoding of scat samples with vertebrate, invertebrate, and plant primers. Additionally, we compared the diets of urban coyotes to those in non-urban areas. This study is the first that we are aware of to use next-generation sequencing (NGS) to examine the diet of any urban carnivore. DNA metabarcoding using NGS amplifies a variable region of DNA to identify species represented in a biological sample such as scat (Porter & Hajibabaei, 2018). NGS can produce hundreds of thousands of DNA sequences for each sample, with many samples examined at the same time (Shendure & Ji, 2008). DNA metabarcoding can identify highly diverse diets, including both prey species and anthropogenic food sources. Vertebrates consumed by coyotes can be identified to the species taxonomic level based on scat DNA, which is not always possible with morphological analysis. Furthermore, in studies comparing both techniques, NGS detected more prey items than morphological analysis (Mumma et al., 2016; Gosselin, Lonsinger & Waits, 2017; Oja et al., 2017). However, metabarcoding may also detect items that attach to the scat post-defecation, such as pollen, seeds, or urine. By using metabarcoding to study the urban coyote diet we can find out whether the coyotes rely on human associated foods or whether they can survive on natural food items.

Here, we hypothesized that coyote diets in urban areas would be less diverse than non-urban coyotes due to proportionally fewer prey species inhabiting urban areas. We also predicted that urban coyotes would consume more human food due to increased access to human refuse in urban areas. In addition, we predicted that deer would make up less of the urban diet because deer are only found in a few small parts of NYC (City of New York, 2020a). Furthermore, we hypothesized that metabarcoding analysis would allow us to detect more species of diet items than morphological analysis.

## Methods

### Sample collection and storage

**Figure 1:**
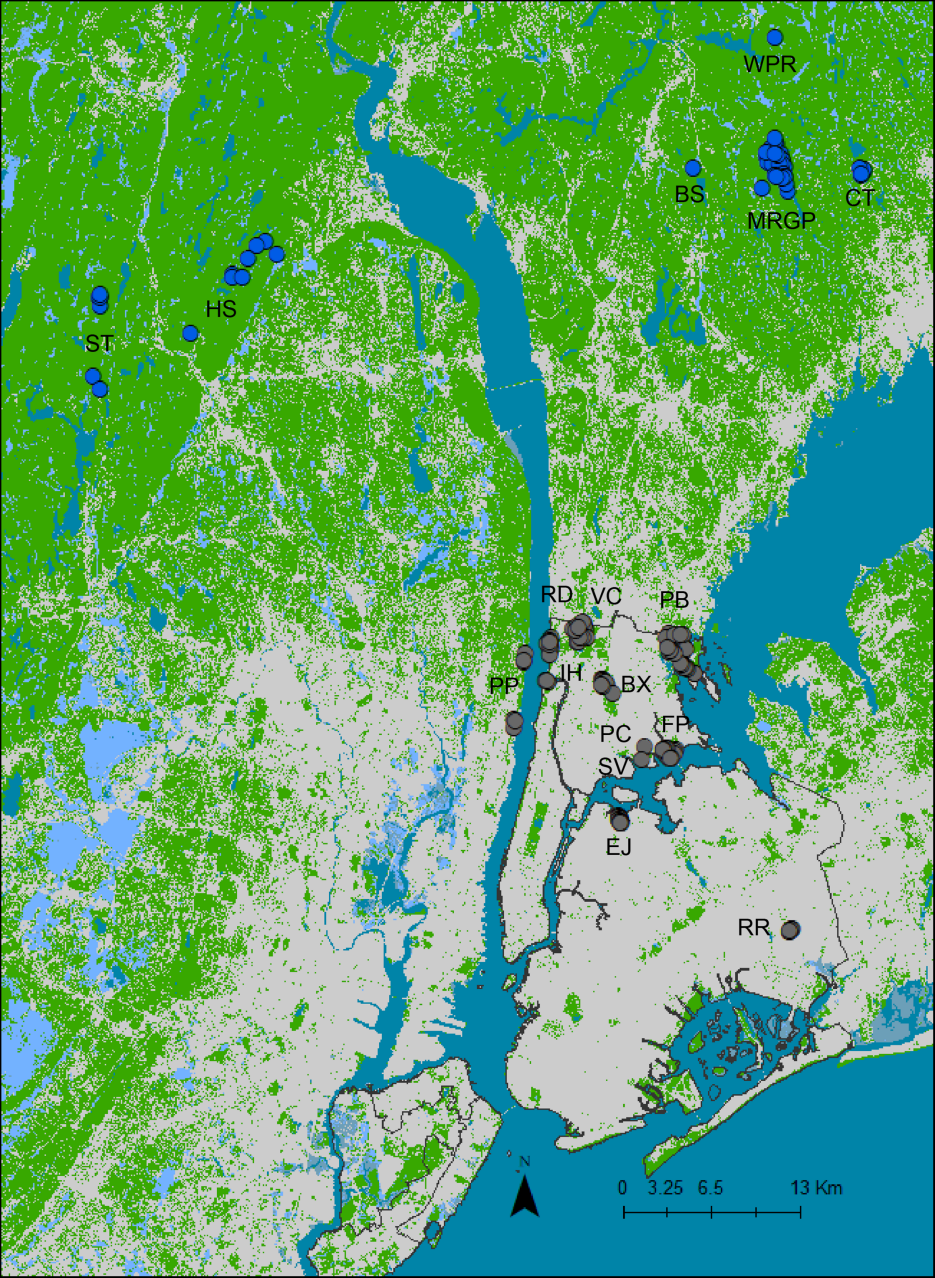
A map of sampling locations created in ArcGIS 10.7.1 (ESRI, 2019) using the 2016 National Land Cover Database (Wickham et al., 2014). Areas in green indicate Deciduous, Evergreen, and Mixed Forests, Developed Open Space, Barren Land, Shrub, Grassland, Pasture/Hay, and Cultivated Crops. Areas in grey depict Low, Medium, and High Intensity Development. New York City is outlined in black. Each sample location is displayed as a circle, with urban locations represented with grey circles and non-urban locations with blue circles. The abbreviations represent the locations surveyed for this study (BS, Butler Sanctuary; BX, Bronx Park; EJ, Elmjack Ballfield; FP, Ferry Point Park; HS, Harriman State Park; IH, Inwood Hill Park; MRGP, Mianus River Gorge Preserve; PC, Pugsley Creek Park; PP, Palisades Parkway; PB, Pelham Bay Park; RD, Riverdale Park; RR, Railroad Park; SF, Stamford Residence; ST, Sterling Forest State Park; SV, Soundview Park; VC, Van Cortlandt Park; WPR, Ward Pound Ridge Reservation).

We used fecal samples (scats) to examine the diets of urban and non-urban coyotes. Scat sampling was opportunistic and occurred between 2007–2017. Urban scat samples were collected at 10 New York City and New Jersey municipal parks and one recreational facility (Fig. 1). Scat collection at NYC parks was approved under NYS Permit #1118. Non-urban samples were collected on hiking trails at five New York state parks and preserves, which are primarily made of hardwood forests (Nagy et al., 2011; New York State Park Service, 2019). Samples were also opportunistically collected at a residence in Stamford, CT. All non-urban sites were located in Westchester, Orange, and Rockland Counties, all situated north of NYC. The parks and preserves were surrounded by suburban and exurban development. No permit was necessary to sample at the non-urban and New Jersey sites because we were not collecting tissue or trapping animals. Dry scats were collected whole and stored in paper bags containing silica desiccant. Samples that were moist upon collection were stored in plastic bags and frozen within 24 h at −20 °C. We used coyote and house fly (*Musca domestica*) tissue samples as positive controls for the vertebrate and invertebrate PCRs, respectively. Detailed information about scat and coyote tissue collection methodology is described in Henger et al. (2019).

### Urbanization category

We define urban as areas containing considerably more impervious surface than other areas (see *Urbanization and Anthropogenic Food* below). To categorize our samples into urban and non-urban, we used the NLCD 2016 Percent Developed Imperviousness data (Xian et al., 2011) to calculate the percentage of impervious surface around the sample collection sites. We used zonal statistics in ArcGIS 10.7.1 to estimate the percent impervious surface at 5 and 10 km buffers around each sample (Kays, Gompper & Ray, 2008). We considered samples with 30–80% impervious surface as urban and those with an 0–10% impervious surface as non-urban, (Fig. S1). No sites contained between 10–30% impervious surface. We calculated the correlation between the percent impervious surface and the count of anthropogenic food items for both the 5 and 10 km buffers using the corr.test() function in the R package “psych”, version 1.8.12 (Revelle, 2022), specifying the Kendall method for non-normal distribution of data. We also calculated the correlation between the percent impervious surface and the percentage of reads of anthropogenic items. In addition, to check whether anthropogenic food consumption is driven by a small number of coyotes, we compared the counts of anthropogenic items found in the scats of coyotes that were individually identified in a previous study (Henger et al., 2019).

### DNA extraction

We extracted DNA from 127 urban scat samples, 59 non-urban scat samples, and three positive PCR controls with the Qiagen DNeasy Blood and Tissue Kit (Qiagen Inc., Hilden, Germany) and modified extraction protocol where we used ethanol that we stored in the freezer for 1 h and we heated the AE buffer at 70 °C before eluting the DNA. We eluted with 100 µl AE buffer by performing two rounds of pipetting 50 µl of heated AE buffer to the DNeasy membrane, incubating at ambient temperature for 30 min, and centrifuging for 3 min at 8,000 rpm. Prior to extraction, we homogenized the scat (hand-mixing it from outside of the collection bag for 10–15 s) and sampled scat fragments from 3–4 different areas of the scat to increase the chance that DNA from all food items would be extracted (Gosselin, Lonsinger & Waits, 2017). We did not use the Qiagen Stool Mini Kit (Qiagen Inc., Hilden, Germany) because the InhibitEX tablets contain potato DNA (Valentini et al., 2009) that might interfere with our plant DNA results. Samples were extracted in groups of 10–12 collected from the same sites or from sites that were located close together. We quantified total DNA concentration with a Qubit 2.0 Fluorometer (Invitrogen, Waltham, MA, USA). We excluded 10 urban samples and five non-urban samples from future analyses because the DNA concentrations were below 2.0 ng/µl.

### Species identification

To distinguish coyote scat from domestic dog, red fox, or other wildlife species, we PCR-amplified the samples with the dye-labelled (6FAM) forward primer SIDL and two different reverse primers, H16145 and H3R (De Barba et al., 2014) that amplify the mitochondrial DNA control region and have a variable fragment number and size across species. The 25 µl PCR consisted of 12.5 µl Qiagen Multiplex PCR Master Mix, 2.5 × Qiagen Q solution, 1 µl SIDL, 1 µl H16145, 1 µl µM H3R, 2 µl DNA extract, and 5 µl sterile water. PCR thermocycler conditions included a 15-min denaturation at 94 °C, followed by 35 cycles of 30 s at 94 °C, 90 s at 46 °C, 60 s at 72 °C, and a 30-min elongation at 60 °C. Samples that exhibited no amplification were PCR-amplified with a second set of primers, KFSPID-F and KFSPID-R (Bozarth et al., 2010). The 25 µl PCR contained 12.5 µl Platinum Taq (ThermoFisher Scientific, Waltham, MA, USA), 1 µl KFSPID-F, 1 µl KFSPID-R, 2 µl DNA extract, and 8.5 µl sterile water. PCR thermocycler conditions included a 10 min denaturation at 96 °C, followed by 35 cycles of 60 s at 94 °C, 60 s at 53 °C, 90 s at 72 °C, and a 5 min elongation at 72 °C. PCR products were sent to Genewiz (Frederick, MD) for fragment analysis and genotypes were assigned using Geneious with the microsatellite plugin.

### Library preparation

We PCR-amplified the 117 urban and 54 non-urban coyote samples with using universal vertebrate (12SV5) (Riaz et al., 2011), invertebrate (16MAV) (De Barba et al., 2013), and plant (trnL) (Taberlet et al., 2007) primers to identify potential food items present in coyote scats. A subset of samples were PCR amplified twice with the vertebrate primer set and the amplified PCR products of both reactions were sequenced and the results of both sequencing runs were used to identify the diets for those samples. Time and financial constraints prevented us from amplifying all samples twice with each of the three primer sets. The range of amplified fragments was 36–98 bp. Our amplicon sizes were too short (<300 bp) to use the Nextera XT Library Prep Kits (Illumina Inc., San Diego, CA, USA) to attach the Illumina adapters to the primers. Instead, we created primers that consisted of the universal primers attached to the ends of the Illumina adapter sequences (Table 1). To prevent the 12SV5 primers from amplifying coyote DNA, we developed a blocking primer, CanisB, that is modified to prevent elongation during PCR and binds to the associated sequence of coyote DNA to prevent amplification (Vestheim & Jarman, 2008) (Table 1). We also used the blocking primer MammMAVB1 to prevent mammal DNA from amplifying with the invertebrate primers 16SMAV-F and 16SMAV-R (De Barba et al., 2013). We ran three 25 µl amplicon PCRs for each sample, each using a different primer pair (vertebrate, invertebrate, plant), using both positive and negative PCR controls. The positive controls were coyote (*C. latrans* tissue; vertebrate), wheat germ (*Triticum aestivum*; plant), and whole house fly (*M. domestica*; invertebrate). We included 30 negative PCR controls (11 vertebrate, 11 plant, 8 invertebrate). The vertebrate and invertebrate PCRs consisted of 12.5 µl Kapa HiFi HotStart ReadyMix (Kapa Biosystems, Wilmington, MA, USA), 1 µl F/R primer, 5.0 µL of blocking primer, 2.0 µl DNA and 4.5 µl sterile water. The plant PCRs included 12.5 µl Kapa Hifi HotStart ReadyMix, 1 µl F/R primer, 2.0 µl DNA and 9.5 µl sterile water. All forward and reverse primers were at concentrations of 10 µM and the blocking primers were at concentrations of 20 µM. The PCR profile had an initial denaturation step of 3 min at 95 °C, followed by 45 cycles of 30 s at 94 °C, 90 s at 55 °C, and no elongation. The PCR products were purified using a Sera-Mag Magnetic Speed-bead mix (Rohland & Reich, 2012) at a volume of 1.8x Sera-Mag beads to PCR product. We used MiSeq Reagent Kit v2 (2 × 150 bp). The Nextera XT Index kit v2 Sets A, C, and D (Illumina Inc., San Diego, CA, USA) were used to label each PCR product with a unique sequence for individual identification. We generated sequencing reads from the scat libraries using two runs of an Illumina MiSeq sequencer (Table S1). The first was at the Bioinformatics and Computational Genomics Laboratory, Hunter College, City University of New York and contained 269 pooled PCR products. The second run was performed at Genewiz Inc., and contained 270 pooled PCR products.

**Table 1:** The primers used to amplify vertebrate, invertebrate, and plant DNA (De Barba et al., 2013).

Taxon	DNA region	Primer name	Forward/reverse/blocking	Sequence (5′-3′)
Vertebrates	12S mtDNA	12SV5-F	Forward	GTCTCGTGGGCTCGGAGATGTGTATAAGAGACAGTTAGATACCCCACTATGC
		12SV5-R	Reverse	TCGTCGGCAGCGTCAGATGTGTATAAGAGACAGTAGAACAGGCTCCTCTAG
		CanisB	Coyote	CCACTATGCTTAGCCCTAAACATAGATAATTTTACAACA-C3
			Blocking	
Invertebrate	16S mtDNA	16SMAV-F	Forward	GTCTCGTGGGCTCGGAGATGTGTATAAGAGACAGCCAACATCGAGGTCRYAA
		16SMAV-R	Reverse	TCGTCGGCAGCGTCAGATGTGTATAAGAGACAGARTTACYNTAGGGATAACAG
		MammMAVB1	Mammal	CCTAGGGATAACAGCGCAATCCTATT-C3
			Blocking	
Plant	*trn*L (UAA)	ITS1-F	Forward	GTCTCGTGGGCTCGGAGATGTGTATAAGAGACAGGGGCAATCCTGAGCCAA
		ITS2-R	Reverse	TCGTCGGCAGCGTCAGATGTGTATAAGAGACAGCCATTGAGTCTCTGCACCTATC

**Note:**

The CanisB blocking primer was identified by this study.

### Sequence analysis and filtering

The first and second sequencing runs contained 12,757,690 and 12,993,587 reads, respectively. The results from both runs were combined and analyzed. We used the ecoPCR program, version 1.0.1 (Ficetola et al., 2010) to generate reference databases for vertebrate, invertebrate, and plant sequences. The ecoPCR program uses *in silico* PCR to identify sequences from the European Molecular Biology Laboratory (EMBL) genetic database that would be amplified with the associated primer sets. We used the OBITOOLS software, version 1.2.11 (Boyer et al., 2016) to align sequences using the *illuminapairedend* command. Next, we used the cutadapt program (Martin, 2011) to trim the forward and reverse primers from the aligned sequences. We then employed the OBITOOLS command *obiannotate* to add the individual sample names to each sample. Afterwards, all sequences from each sample were concatenated into one file. We used the *obiuniq* command to merge duplicate sequences and to remove low quality sequences from further analysis (alignment quality <40, read count <10, vertebrate sequence length <80 bp and >150 bp, plant sequence length <30 bp and >80 bp, invertebrate sequence length <15 bp and >80 bp). We employed the *obigrep* command to remove sequences that were unaligned. We used the *ecotag* command in OBITOOLS to match the sequences to each of the reference databases, and then filtered out sequences that were lower than a 98% match to the vertebrate and plant reference databases and lower than a 97% match to the invertebrate database. To filter out environmental contamination and PCR artifacts, we only considered items as part of the diet if they comprised at least 1% of the total vertebrate reads (Elfström et al., 2014) and 5% of the total plant and invertebrate reads and were present in the sample at 500 copies or more.

For some of the diet items, it was unclear whether they were found naturally or as human food. We placed such ambiguous items in the most likely category depending on the natural distribution of the taxa. For example, the grape sequences could be from wild grape plants or from grapes eaten as anthropogenic food. We categorized grape as a plant instead of human food because several species of grapevine grow throughout New York and are found in the parks where we sampled the scats (New York Flora Association, 2019). Similarly, we included turkey as a natural food item because turkeys inhabit parks and other green spaces within NYC and throughout New York state (City of New York, 2020b). In contrast, we categorized chicken and pig as human food because those species are common as human food but are not naturalized in NYC parks. We were unable to detect domestic dog in the diet because coyotes and dogs have the same genetic sequence at the 12SV5 region of the mitochondrial genome. In addition, taxonomy assignment is restricted to local species. For example, white-tailed deer shares the same nucleotide sequence in the 12SV5 region as mule deer, but only white-tailed deer live in the Northeastern United States.

We grouped the diet items into eight categories: small mammals, other mammals, white-tailed deer, birds, plants, insects, aquatic, and anthropogenic. We chose similar categories as those used in previous coyote diet research (cited in Introduction) to better enable comparisons between studies. In general, those coyote diet studies grouped diet items into mammals, birds, invertebrates, plants, and anthropogenic food. However, they differ in how they subdivide the mammal section into small groups. We chose to use “small mammals” and “other mammals” to differentiate small prey such as rodents and rabbits from larger prey items including raccoon and beaver. We added an “aquatic” category to reflect the prey items that live in or near the water, such as salamander and fish, and were not already categorized by any of the other groups. For example, the genus *Anas* (dabbling ducks) is listed in the bird category instead of the aquatic category. We report the coyote diet as frequency of occurrence (FO) calculated as FO_*i*_(%) = (n_i_/N) * 100, where n_i_ represents the number of scats containing the diet item and N is the total number of scats. We also report the relative frequency of occurrence for each diet category, calculated as the number of occurrences of each food item in the category divided by the total number of food items.

### Diet diversity analyses

We compared the diet species diversity between the urban and non-urban coyotes using the Shannon-Weiner index with the R package “vegan, version 2.5.7” (Oksanen et al., 2019). We calculated the diversity at the molecular operational taxonomic unit (MOTU) level. We standardized for uneven sampling sizes between the two groups by dividing the total count of each species by number of individual coyotes included in the group. Statistically significant differences in Shannon diversity indices were examined using Hutchinson’s t-test (Hutcheson, 1970) in the R package “ecolTest, version 0.0.1” (Salinas & Ramirez-Delgado, 2021). We used the R package “EcoSimR, version 0.1.0” (Gotelli, Hart & Ellison, 2015) to calculate Pianka’s niche overlap indices (Pianka, 1973) in resource categories between the two groups of coyotes. The indices range from 0–1, where 0 indicates no overlap and 1 complete overlap. To investigate whether differences in the dietary species richness (the number of unique diet items) of the two groups was solely due to disparity between the sample sizes of scats, we performed a rarefaction analysis using the R package iNEXT, version 2.0.20 (Chao et al., 2014). The rarefaction analysis extrapolates the number of diet items detected with increased sample sizes. In addition, we calculated the correlation between percentage of impervious surface and the percentage of reads from anthropogenic items in each sample. We used the ANOSIM test (Clarke, 1993; Warton, Wright & Wang, 2012) in the R package “vegan, version 2.5.7” to test for a significant difference between the diets of urban and non-urban coyotes. To visualize the difference in diet composition between the two groups, we used the R package “FactoMineR, version 1.41” (Le, Josse & Husson, 2008), to perform a Principal Components Analysis (PCA) and “factoextra”, version 1.0.5 (Kassambara & Mundt, 2017) to generate a PCA biplot. A PCA reduces many correlated variables into a few variables that are uncorrelated with each other. A biplot displays the results of a PCA along with a representation of the degree to which each variable is associated with the samples. The SIMPER test (Clarke, 1993) in the R package “vegan, version 2.5.7” was used to determine which diet categories contributed to the differences between the diets.

### Methodological comparison

We compared our metabarcoding analysis to a morphological analysis of the same scat samples (Duncan et al., 2020). The two diet studies shared 68 urban samples (Table S2). We calculated the Jaccard Similarity Index (Jaccard, 1912) between the two datasets using the equation: J(X,Y) = |X∩Y|/|X∪Y|, comparing the number of diet items shared between the two datasets (the item was detected in the same sample by both methodologies) to the total number of items. We hypothesized that the Jaccard similarity between the two methodologies would be close to 1, reflecting very similar results. However, we expected to detect more items with the genetic analysis than the morphological analysis because the genetic analysis should be able to amplify DNA from human food as well as DNA that remains in the stomach after morphologically identifiable remains have been excreted. Therefore, we also used Jaccard Similarity Index to compare only the diet items detected by morphological analysis that were also detected by metabarcoding. Additionally, we compared the Jaccard Similarity Index of different diet categories (mammals, plants, invertebrates, anthropogenic) to determine whether either method was better than the other at detecting diet items within certain categories. Due to differences in the sensitivities of the two methodologies, we compared mammals at the Species taxonomic level, but for the rest of the diet items we only compared the number of scats that contained any birds, plants, invertebrates, and anthropogenic food items.

## Results

Among urban samples, we identified 110 coyotes, no red foxes or domestic dog, and seven samples exhibited no amplification. Out of 110 urban coyote samples, 80 amplified with the vertebrate primers, 35 amplified with the invertebrate primers, and 77 amplified with the plant primers, and 15 did not amplify with any of the primers. Among non-urban samples, we identified 32 coyotes, 15 red foxes, three domestic dog, and four samples exhibited no amplification. Our final dataset consisted of 95 urban scats and 31 non-urban scats. The vertebrate and plant sequences could all be identified to Family, and most of the vertebrates could be identified to genus and species. Invertebrate sequences could be identified to order.

Amplification was observed in all 11 vertebrate, six plant, and two invertebrate PCR negative controls. The vertebrate species amplified in the negative controls were pigeon/dove, pig, chicken, and turkey. The plant families amplified were Poaceae, Fabaceae, Fagaceae, Oleaceae, Juglandaceae, Rosaceae, Musaceae, and Salicaceae. The order Diptera was the only invertebrate order amplified in the negative controls. We removed any sequences from the samples comprising the corresponding PCR batch that were identical to those detected in the PCR negative controls.

Both urban and non-urban coyotes consumed a variety of mammals and plant species, as well as anthropogenic food items (Table 2). Raccoons were one of the most common food items detected in the scat of both urban and non-urban coyotes (27% and 48% of scats, respectively). Urban coyotes consumed more species of birds than non-urban coyotes (10 bird species compared to two species in the non-urban diet). Many of the birds consumed by urban coyotes were from three taxonomic groups commonly found in urban environments: *Anas* (dabbling ducks), Columbidae (pigeons and doves), and Sturnidae (European starlings).

**Table 2:** The count of scats that contained each diet item from the metabarcoding analysis. The frequency of occurrence (FO) is listed in parentheses. The total count and FO are also listed for each of the eight diet categories. The following representative silhouettes of groups were obtained from phylopic.org. Small Mammals image (mouse) courtesy of Anthony Caravaggi under the Attribution-NonCommerccial ShareAlike 3.0 Unported license, https://creativecommons.org/licenses/by-nc-sa/3.0/. Birds image (pigeon) (http://phylopic.org/image/a62a398d-793c-48cf-9803-e52118a28639/) courtesy of Luc Viatour under the Attribution-ShareAlike 3.0 Unported license, https://creativecommons.org/licenses/by-sa/3.0/. Other Mammals image (raccoon) courtesy of Mathieu Basille (http://phylopic.org/image/e805d164-21e7-4657-979a-226f6ccc7f15/), Deer image courtesy of Oscar Sanisidro (http://phylopic.org/image/bb553480-e37f-4236-8c69-ce9fa8116b39/), and Aquatic image (salamander) courtesy of zoosnow (http://phylopic.org/image/d1644001-d86d-4541-9501-295a873aed2a/), all under the Public Domain Dedication 1.0 license, https://creativecommons.org/publicdomain/zero/1.0/. Plants image (grass) is uncredited (http://phylopic.org/image/2af0a13e-69a8-4245-832e-ee3d981089b7/) and the Insects image (cricket) (http://phylopic.org/image/b80d830b-155a-4ca5-9119-9a9fde019cc6/) courtesy of Thomas Hegna are both under the Public Domain Mark 1.0 license, https://creativecommons.org/publicdomain/mark/1.0/. The Anthropogenic image (drumstick) was obtained from https://www.vectorstock.com/royalty-free-vector/fried-chicken-drumstick-vector-1166263 courtesy of Tribaliumvs, under the standard license.

Scientific name (Common name)	Count (Urban)	Count (Non-Urban)
Small mammals 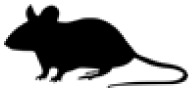	33 (34.7%)	11 (35.48%)
*Peromyscus leucopus* (white-footed mouse)	2 (2.1%)	3 (9.7%)
*Rattus norvegicus* (brown rat)	5 (5.3%)	0 (0%)
*Blarina brevicauda* (northern short-tailed shrew)	1 (1.1%)	0 (0%)
*Sylvilagus* (rabbit)	13 (13.7%)	4 (12.9%)
*Microtus pennsylvanicus* (meadow vole)	12 (12.6%)	4 (12.9%)
*Sciurus carolinensis* (eastern grey squirrel)	3 (3.2%)	2 (6.5%)
*Tamias striatus* (eastern chipmunk)	3 (3.2%)	2 (6.5%)
*Ondatra zibethicus* (muskrat)	5 (5.3%)	0 (0%)
Other mammals 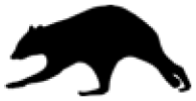	30 (31.6%)	16 (51.6%)
*Lynx rufus* (bobcat)	1 (1.1%)	1 (3.2%)
*Castor canadensis* (North American beaver)	0 (0%)	1 (3.2%)
*Didelphis virginiana* (Virginia opossum)	2 (2.1%)	0 (0%)
*Marmota monax* (groundhog)	0 (0%)	2 (6.5%)
*Vulpes vulpes* (red fox)	1 (1.1%)	2 (6.5%)
*Procyon lotor* (raccoon)	26 (27.4%)	13 (41.9%)
*Mephitis mephitis* (striped skunk)	2 (2.1%)	0 (0%)
*Odocoileus virginianus* 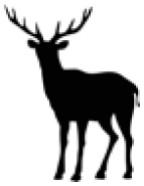		
(white-tailed deer)	9 (9.5%)	13 (41.9%)
Birds 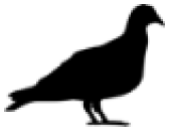	26 (27.4%)	10 (32.3%)
Accipitridae (bird of prey)	1 (1.1%)	0 (0%)
*Anas* (dabbling duck)	8 (8.4%)	0 (0%)
*Corvus brachyrhynchos* (American Crow)	1 (1.1%)	0 (0%)
*Leucophaeus atricilla* (laughing gull)	1 (1.1%)	0 (0%)
Turdidae (Thrush)	1 (1.1%)	0 (0%)
Anatidae (swan, goose, duck)	2 (2.1%)	0 (0%)
Columbidae (pigeon and dove)	9 (9.5%)	4 (12.9%)
*Catharus ustulatus* (Swainson’s thrush)	2 (2.1%)	0 (0%)
*Sturnus vulgaris* (European starling)	5 (5.3%)	0 (0%)
*Meleagris gallopavo* (turkey)	9 (9.5%)	6 (19.4%)
Plants 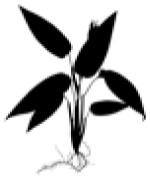	76 (80.0%)	22 (71.0%)
Anacardiaceae (sumac)	4 (4.2%)	0 (0%)
Asteraceae (aster)	36 (37.9%)	2 (6.5%)
Celastraceae (Staff-vine)	0 (0%)	2 (6.5%)
Convolvulaceae (morning glory)	2 (2.1%)	0 (0%)
Fabaceae (legume)	5 (9.5%)	0 (0%)
Fagaceae (beech)	14 (14.7%)	12 (38.7%)
Juglandaceae (walnut)	12 (12.6%)	5 (5.3%)
*Morus* (mulberry)	4 (4.2%)	0 (0%)
*Pinus* (pine)	1 (1.1%)	0 (0%)
Poaceae (grass)	19 (20.0%)	3 (9.7%)
*Vitis* (grape)	13 (13.7%)	1 (3.2%)
Rosaceae (rose)	24 (25.3%)	7 (22.6%)
Salicaceae (willow)	12 (12.6%)	0 (0%)
*Solanum* (nightshade)	2 (2.1%)	0 (0%)
Apiaceae (parsley)	2 (2.1%)	0 (0%)
Oleaceae (ash)	0 (0%)	3 (9.7%)
*Prunus* (wild cherry)	3 (3.2%)	1 (3.2%)
Insects 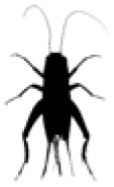	32 (33.7% )	7 (22.6%)
Gryllidae (cricket)	2 (2.1%)	0 (0%)
Diptera (fly, mosquito, gnat, midge)	23 (24.2%)	5 (16.1%)
Coleoptera (beetle)	10 (10.5%)	3 (9.7%)
Lepidoptera (butterflies and moths)	1 (1.1%)	2 (6.5%)
Aquatic 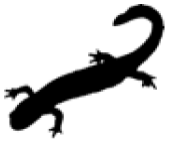	7 (7.4%)	1 (3.2%)
*Desmognathus fuscus* (dusky salamander)	3 (3.2%)	1 (3.2%)
*Lepomis* (sunfish)	1 (1.1%)	0 (0%)
Penaedae (prawn)	2 (2.1%)	0 (0%)
Decapoda (shrimp, crab, lobster)	1 (1.1%)	0 (0%)
Anthropogenic 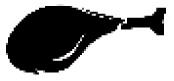	61 (64.2%)	17 (54.8%)
*Capra* (goat)	3 (3.2%)	0 (0%)
*Ovis* (sheep)	1 (1.1%)	0 (0%)
*Oryza* (rice)	5 (5.3%)	0 (0%)
*Bos Taurus* (cow)	4 (4.2%)	0 (0%)
*Pyrus* (pear)	1 (1.1%)	0 (0%)
*Glycine* (soybean)	4 (4.2%)	0 (0%)
*Felis catus* (domestic cat)	4 (4.2%)	0 (0%)
*Gallus gallus* (chicken)	46 (48.4%)	14 (45.2%)
*Musa* (banana)	2 (2.1%)	0 (0%)
*Sus scrofa* (pig)	17 (17.9%)	4 (12.9%)
*Thunnus* (tuna)	1 (1.1%)	0 (0%)
*Pisum* (pea)	2 (2.1%)	0 (0%)
*Numida meleagris* (guineafowl)	3 (3.2%)	0 (0%)
Total	387	138

**Figure 2:**
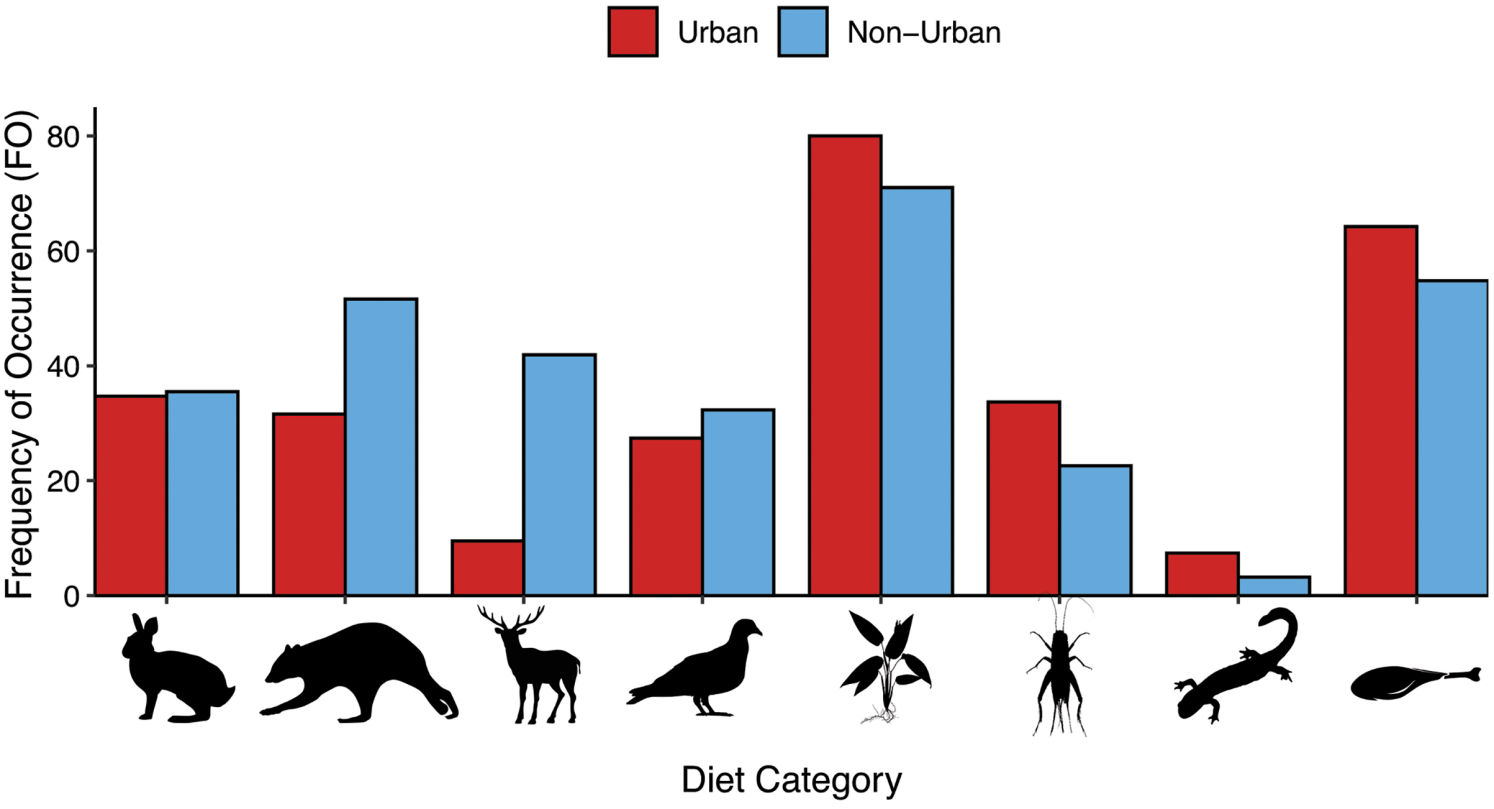
A comparison of the frequency of occurrence (FO) of diet items detected in the urban (*n* = 95) and non-urban (*n* = 31) coyotes. From left to right, categories are small mammals, other mammals, deer, birds, birds, plants, insects, aquatic, and anthropogenic. Urban bars are located on the left (red) and non-urban bars are on the right (blue). The following representative silhouettes of groups were obtained from phylopic.org. Small Mammals image (mouse) courtesy of Anthony Caravaggi under the Attribution-NonCommerccial ShareAlike 3.0 Unported license, https://creativecommons.org/licenses/by-nc-sa/3.0/. Birds image (pigeon) (http://phylopic.org/image/a62a398d-793c-48cf-9803-e52118a28639/) courtesy of Luc Viatour under the Attribution-ShareAlike 3.0 Unported license, https://creativecommons.org/licenses/by-sa/3.0/. Other Mammals image (raccoon) courtesy of Mathieu Basille (http://phylopic.org/image/e805d164-21e7-4657-979a-226f6ccc7f15/), Deer image courtesy of Oscar Sanisidro (http://phylopic.org/image/bb553480-e37f-4236-8c69-ce9fa8116b39/), and Aquatic image (salamander) courtesy of zoosnow (http://phylopic.org/image/d1644001-d86d-4541-9501-295a873aed2a/), all under the Public Domain Dedication 1.0 license, https://creativecommons.org/publicdomain/zero/1.0/. Plants image (grass) is uncredited (http://phylopic.org/image/2af0a13e-69a8-4245-832e-ee3d981089b7/) and the Insects image (cricket) (http://phylopic.org/image/b80d830b-155a-4ca5-9119-9a9fde019cc6/) courtesy of Thomas Hegna are both under the Public Domain Mark 1.0 license, https://creativecommons.org/publicdomain/mark/1.0/. The Anthropogenic image (drumstick) was obtained from https://www.vectorstock.com/royalty-free-vector/fried-chicken-drumstick-vector-1166263 courtesy of Tribaliumvs, under the standard license.

The coyotes inhabiting urban areas consumed proportionally more plants, anthropogenic food items, and insects than the non-urban coyotes, but fewer deer, other mammals, and birds than non-urban coyotes (Table 2, Fig. 2). Both groups of coyotes ate similar proportions of small mammals and aquatic species (dusky salamander, sunfish, prawn, and decapods). Bobcat was detected in both urban and non-urban scats and red fox was only found in non-urban scats. Urban coyotes ate a variety of human foods, including meat (pork, beef, goat, sheep, chicken), fruit and vegetables (chickpeas, olives, pear, banana), and rice, whereas non-urban coyotes only consumed chicken and pork. Chicken was the most detected human food item in the urban and non-urban coyote scats (Table 2).

**Figure 3:**
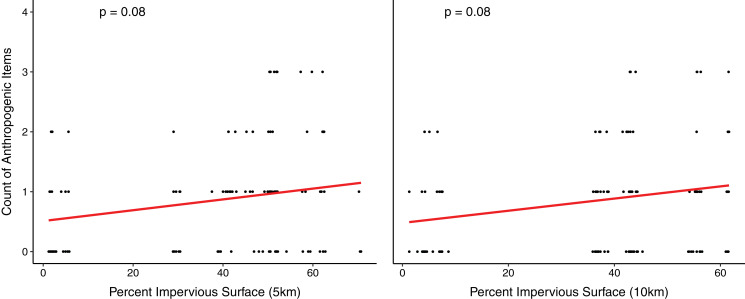
The number of anthropogenic food items detected in coyote scat at varying levels of impervious surface in New York.

Urban coyotes consumed more species of anthropogenic food (13 compared to two) than non-urban coyotes, but they had similar proportions of it in their diets (urban FO = 64.2%, non-urban FO = 54.8%). We found a slight correlation between the count of anthropogenic items consumed and the percent impervious surface at both a 5 km buffer (*p* = 0.08) and a 10 km buffer (*p* = 0.08) (Fig. 3), but it was not statistically significant. Similarly, the correlation between percent of impervious surface and percentage of reads from anthropogenic items was not statistically significant at a 5 km buffer (*p* = 0.06) and a 10 km buffer (*p* = 0.06) (Fig. S2). To determine whether anthropogenic food consumption was driven by only a small number of coyotes, we analyzed the diets of individuals who could be differentiated by unique microsatellite genotypes (Henger et al., 2019). We found that 57 of the 94 urban scats were deposited by 25 individuals. We detected anthropogenic food in scats of 22 of those 25 individuals (88%). Domestic cats were detected in 4.2% of the urban scats and were not found in non-urban scats.

**Figure 4:**
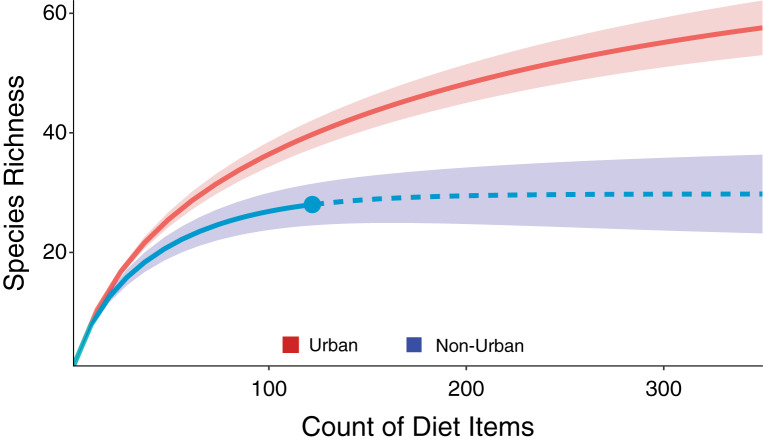
A rarefaction analysis to compare the species richness of diet items consumed in the urban (top, red) and non-urban (bottom, blue) coyote groups. The non-urban group would still have a less species-rich diet than the urban group even if more samples were used in the analysis.

**Figure 5:**
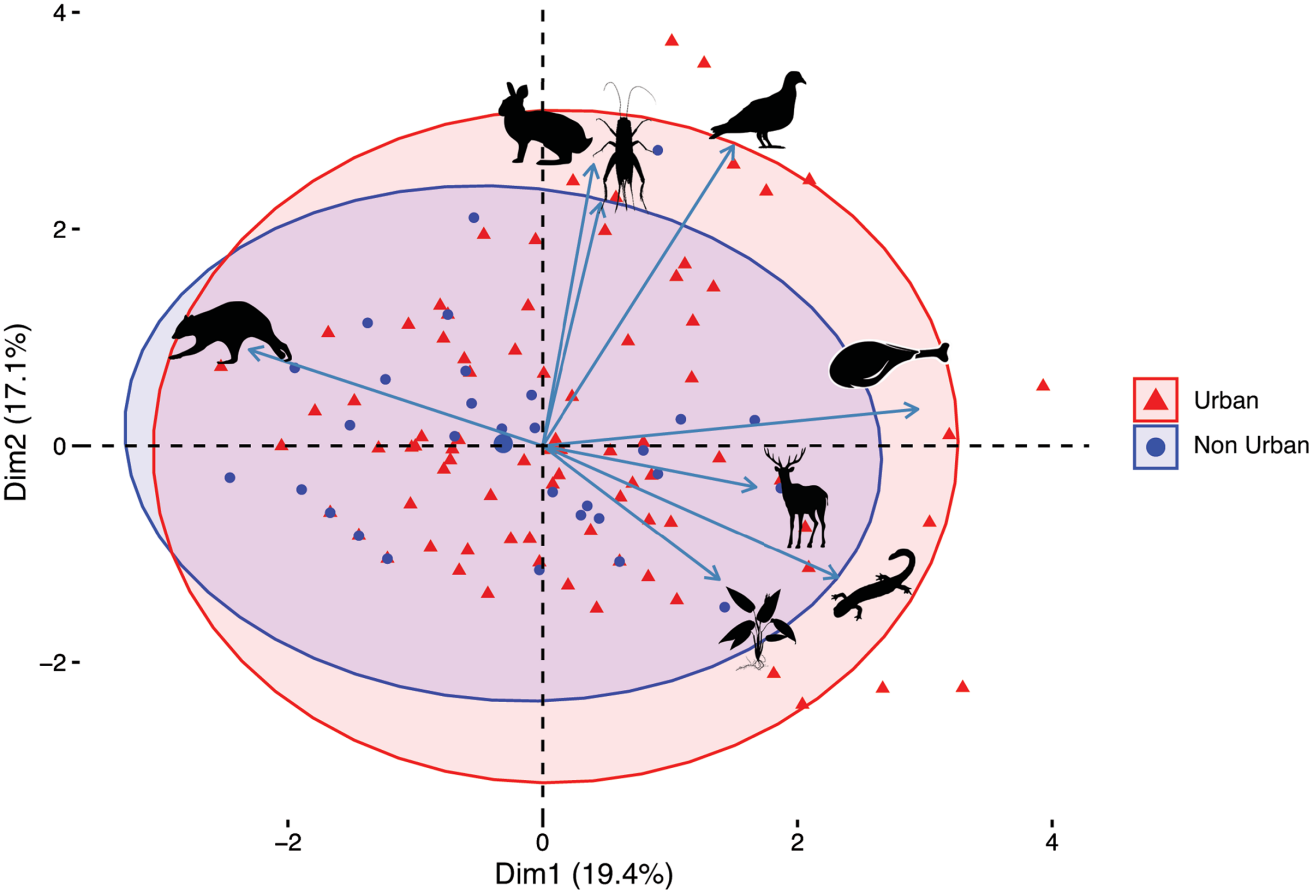
PCA biplot comparing categories of diet items consumed by the urban (red triangles) and non-urban (blue circles) coyote groups. Each point represents one sample and ellipses surround the majority of sample points in each group. Blue arrows indicate the degree to which the diet categories are associated with the variation in the data. Clockwise from the top, the categories are small mammals, insects, birds, anthropogenic, deer, aquatic, plants, and other mammals. The following representative silhouettes of groups were obtained from phylopic.org. Small Mammals image (mouse) courtesy of Anthony Caravaggi under the Attribution-NonCommerccial ShareAlike 3.0 Unported license, https://creativecommons.org/licenses/by-nc-sa/3.0/. Birds image (pigeon) (http://phylopic.org/image/a62a398d-793c-48cf-9803-e52118a28639/) courtesy of Luc Viatour under the Attribution-ShareAlike 3.0 Unported license, https://creativecommons.org/licenses/by-sa/3.0/. Other Mammals image (raccoon) courtesy of Mathieu Basille (http://phylopic.org/image/e805d164-21e7-4657-979a-226f6ccc7f15/), Deer image courtesy of Oscar Sanisidro (http://phylopic.org/image/bb553480-e37f-4236-8c69-ce9fa8116b39/), and Aquatic image (salamander) courtesy of zoosnow (http://phylopic.org/image/d1644001-d86d-4541-9501-295a873aed2a/), all under the Public Domain Dedication 1.0 license, https://creativecommons.org/publicdomain/zero/1.0/. Plants image (grass) is uncredited (http://phylopic.org/image/2af0a13e-69a8-4245-832e-ee3d981089b7/) and the insects image (cricket) (http://phylopic.org/image/b80d830b-155a-4ca5-9119-9a9fde019cc6/) courtesy of Thomas Hegna are both under the Public Domain Mark 1.0 license, https://creativecommons.org/publicdomain/mark/1.0/. The Anthropogenic image (drumstick) was obtained from https://www.vectorstock.com/royalty-free-vector/fried-chicken-drumstick-vector-1166263 courtesy of Tribaliumvs, under the standard license.

Overall, the urban coyotes had a more species-rich diet than the non-urban coyotes (61 species compared to 29). This pattern still holds if anthropogenic sources are not included in the analysis (48 species compared to 27 species). The results of the rarefaction analysis indicate that the non-urban coyotes would still have a lower species-rich diet even if we had included more samples in the analysis (Fig. 4). The ANOSIM test returned a significant difference between the two coyote groups (R = 0.073, *p* = 0.049). The PCA showed that the differences in diets between the urban and non-urban coyotes were mostly driven by more plants and human food in the urban diet and the category of “other mammals” in the non-urban diet (Fig. 5). The difference was reflected in the SIMPER test which listed plants, anthropogenic food, and other mammals as the categories contributing to the most to dissimilarities between the diets (cumulative dissimilarity contribution = 55.8%). The Shannon diversity index was 3.4978 for the urban coyotes and 3.0026 for the non-urban coyotes, but the difference was not significant (*p* = 0.4636). We calculated Pianka’s niche overlap at 0.85.

### Methodological comparison

The relative frequencies of the diet categories analyzed by morphological analysis were 36% mammals, 15% birds, 33% plants, 9% invertebrates, and 7% anthropogenic. In comparison, the metabarcoding analysis returned relative frequencies of 29% mammals, 12% birds, 25% plants, 12% invertebrates, and 22% anthropogenic. When comparing the percentage of diet items detected by morphological analysis that were detected by metabarcoding, the Jaccard similarity was 61.3%. When comparing diet categories between the two methodologies, the Jaccard Similarity Index ranged from 29.6% to 66.2% (Table 3).

**Table 3:** The Jaccard Similarity Index of diet categories between this study and the results of Duncan et al. (2020).

	Mammal (# detections)	Birds (# scats)	Invertebrates (# scats)	Plants (# scats)	Anthropogenic (# scats)
Morphological (Duncan et al., 2020)	70	24	14	54	11
Metabarcoding (This study)	66	19	27	56	49
Jaccard similarity	29.6%	17.6%	24.2%	66.2%	15.1%

**Note:**

Mammals were compared at the species taxonomic level and birds, invertebrates, plants, and anthropogenic items were compared by the number of scats that contained those items.

## Discussion

Both urban and non-urban coyotes consumed a broad range of plants, mammals and anthropogenic food. Raccoons were one of the most common mammals detected in the scats of both groups of coyotes. This finding is in contrast to previous research that has not found raccoons to be a significant part of the coyote diet (Quinn, 1997; Grigione et al., 2011; Crimmins, Edwards & Houben, 2012; Poessel, Mock & Breck, 2017; Larson et al., 2020). Since raccoons have become abundant in urban and suburban areas due to the lack of top-down control by larger predators (Hadidian et al., 2010), coyotes in the New York metropolitan area may be opportunistically preying on a plentiful and widely available resource. However, it is unclear how much the coyotes scavenged, as opposed to predated, raccoons and other mesocarnivores. In San Francisco, CA, raccoons were the most common identified roadkill species (Kreling, Gaynor & Coon, 2019). It is possible that the NYC coyote population scavenged raccoons that had already died from vehicle collisions. Similarly, many of the birds that urban coyotes consumed were from Sturnidae, Columbidae, and *Anas*, which contain species that are very common and abundant in urban areas. It is likely that the urban coyotes opportunistically prey upon or scavenge adult birds and/or their eggs because they are such a plentiful resource.

Deer was one of the main food items detected in the non-urban coyote population (FO = 41.9%). This finding is in accordance with the results of Peterson et al. (2021) which detected deer in 60% of coyote scats in Westchester, New York. Eastern coyotes are larger than coyotes in other areas of the United States, and it is hypothesized that their larger size may help them to hunt deer more effectively (Monzõn, Kays & Dykhuizen, 2014). Studies performed in Pennsylvania and South Carolina found that coyotes are the top predator of fawns (Vreeland, Diefenbach & Wallingford, 2004; Kilgo et al., 2012). Of the non-urban coyote samples that contained white-tailed deer DNA 69.23% were collected from April–June, which overlaps with fawning season in New York (Cheatum & Morton, 1946). Chitwood et al. (2015) found that fawn survival was low enough to cause deer population declines. Other studies, however, concluded that deer populations continued to grow even in the presence of coyotes (Parker, 1995; Bragina et al., 2019). As is possible with raccoons, the presence of deer in the coyote diet does not necessarily indicate that coyotes actively hunted and killed the deer found in their diets. A portion, or even all of the deer detected in the diet, may be the result of coyotes scavenging roadkill deer. Deer was detected in only 9.5% of the urban coyote scats. The low proportion of deer in the urban coyote diet was likely influenced by the relatively few urban parks in our study area known to contain deer. Out of 36 scats collected in the two NYC parks (Pelham Bay and Van Cortlandt) that have deer populations, only seven contained deer (19.4%). Even if deer are available, they are consumed at a lesser rate in urban areas.

We detected red fox in the non-urban diet and bobcat in both the urban and non-urban diets, which may be evidence of competition between urban predators. Coyotes typically exclude red foxes from coyote territory (Gosselink et al., 2003; Lombardi et al., 2017), presumably because of high dietary overlap between the two species (Major & Sherburne, 1987). This competitive exclusion ensures that Eastern coyotes do not overlap with foxes in predation of small mammals. Competitive exclusion has not been found in areas where bobcats and coyotes have territorial overlap, but coyotes have predated on bobcats (Fedriani et al., 2000). Alternatively, it is possible that the coyote scats were contaminated with fox and bobcat urine or that coyotes scavenged road-killed animals.

Urban coyotes had a more species-rich diet than the non-urban coyotes. Fedriani, Fuller & Sauvajot (2001) also found a positive correlation between diet diversity and level of urbanization in California coyotes. They reasoned that the diversity was due to the increased availability of anthropogenic foods. Similarly, in this study, many of the additional species included in the urban diet were anthropogenic food items. The urban parks are surrounded by densely populated neighborhoods with a diverse assortment of restaurants, food trucks, and apartment buildings that could all provide refuse for coyotes. Parks themselves may also be sources of refuse from garbage containers and litter. However, after removing human food items from the analysis, the diet of urban coyotes still contained more species than the non-urban diet. One reason for this finding may be that the urban parks offer a greater number of microhabitats that can accommodate more types of species. The urban coyote diet included more species of mammals, plants, and insects than the non-urban diet. New York City parks include a mixture of wetlands, forests, and meadows (NYC Parks Dept, 2019) whereas New York state parks mostly consist of hardwood forests (Nagy et al., 2011; New York State Park Service, 2019). Additionally, relatively higher numbers of white-tailed deer in non-urban areas may decrease plant species richness and contribute to the lower species diversity (Habeck & Schultz, 2015). Another reason for the more species rich diet of the urban coyotes could be that the prey species’ abundances are lower in urban areas, which requires more frequent prey switching (Randa et al., 2009).

Though the urban coyotes consumed more species of anthropogenic food than the non-urban coyotes, they had similar proportions of it in their diets (64.2% of scats of urban coyotes, 54.8% of non-urban coyote scats). Similarly, we found no correlation between anthropogenic food consumed and the impervious surface associated with each sample. This result differed from previous research showing more anthropogenic food consumption by coyotes inhabiting more urbanized areas (Fedriani, Fuller & Sauvajot, 2001; Morey, Gese & Gehrt, 2007; Newsome et al., 2015; Larson et al., 2020; but see Santana & Armstrong, 2017). It is likely that anywhere that humans and coyotes share the same space, coyotes can find anthropogenic food. However, the proportion of anthropogenic food use does not seem to increase relative to the human population density (Table S3). We also found no indication of anthropogenic food use being driven by just a few coyotes. Instead, anthropogenic food items were detected in 88% of the scats of individually identified coyotes. Diet supplementation with human food appears to be a universal trend among coyotes instead of a behavior performed by a small number of individuals. We detected domestic cats only in the urban coyote scats and they were present in less than 5% of the scats. Studies from Chicago, San Diego, Los Angeles, Tucson, Cleveland, and Albany also found low incidences of cats (≤3%) in the coyote diet (MacCracken, 1982; McClure, Smith & Shaw, 1995; Fedriani, Fuller & Sauvajot, 2001; Cepek, 2004; Morey, Gese & Gehrt, 2007; Bogan, 2012).

### Methodological comparison

The relative frequency of occurrence (RFO) of the diet categories differed between the metabarcoding and the morphological studies. One reason for the difference is that more anthropogenic items were detected with DNA metabarcoding. Metabarcoding may be a more accurate way to infer anthropogenic food use than visual detection of human-associated items. We were able to detect anthropogenic items in the diet that would not have been detected morphologically because usually such items are mostly or entirely digested. When a coyote eats a wild animal, there will be hair, skin, feathers, bones, *etc*. consumed as well as the meat that will persist through the digestive tract and appear in the scat. Anthropogenically-sourced meat (*e.g*., chicken or port) have no or far less persistent body parts since they were prepared (deboned, de-feathered, sliced, *etc*.) for human consumption. Instead of relying on human-associated items, such as plastic and food wrappers to infer anthropogenic food consumption, metabarcoding allowed us to detect the actual species consumed.

The Jaccard Similarity Index between the metabarcoding and morphological analyses was low (30.8%), even when we limited the comparison to evaluate the percentage of food items detected by morphological analysis that were also detected by metabarcoding (66.2%). We found that the results from the two methodologies were most similar in the number of scats containing plants (66.2%) and least similar in the number of scats containing anthropogenic items (15.1%). The metabarcoding analysis detected more invertebrates and anthropogenic food items than the morphological analysis. The counts of mammals, birds, and plants are similar for both methodologies, but they were rarely detected by both metabarcoding and morphological analysis in the same samples. One reason for the low similarity between the two methodologies could be that the scat was not sufficiently homogenized and thus was not amplified by the PCR. Another reason could be that some species were amplified better than others with the universal primers. For example, Pawlucyk et al. (2015) found that the primers for the *matK* and *rbcL* genes detected only 50% and 82% of the total plant species, respectively. It is also possible that fewer species amplified in the more degraded scat samples than the fresher samples (McInnes et al., 2017; Alberdi et al., 2019).

The metabarcoding analysis allowed us to identify most vertebrate prey to Species. This is not always possible with morphological analysis of scats, in which some vertebrates are identified to Family (Murray et al., 2015) or cannot be identified and are categorized as “other mammals” (McClure, Smith & Shaw, 1995), “other vertebrates” (Fedriani, Fuller & Sauvajot, 2001), “other” (Quinn, 1997), or “unknown” (Cepek, 2004; Morey, Gese & Gehrt, 2007). We were also able to identify the different bird species in the diet, which is very difficult with morphological and isotopic techniques. When those taxa are recorded as part of the coyote diet, they are only grouped as “birds” instead of identified to Species (Quinn, 1997; Morey, Gese & Gehrt, 2007; Murray et al., 2015). Additionally, we could detect plants to Family and invertebrates to Order with metabarcoding but could only identify them as “plants” and “insect” in the morphological analysis.

However, the ability to detect species in scat with only microscopic residues present in the fecal material has drawbacks, especially when characterizing a generalist species in which nearly any DNA present potentially represents a legitimate diet item. First, cells of species that end up in or on the scat after it was defecated, from sources such as pollen, seeds, and potentially urine, can be genotyped and cause a form of false positive error. Second, secondary predation, where the prey items of a prey item are genotyped and added as primary items is a real possibility (Tercel, Symondson & Cuff, 2021). For example, if a coyote eats a raccoon that had previously eaten chicken from a garbage can, both the chicken and raccoon may be genotyped and added to the coyote’s diet assemblage. Third, metabarcoding is unable to differentiate between prey items that were directly predated *vs* those that were scavenged; while both are legitimate diet items in terms of characterizing what the target species is eating overall, this distinction is often important to ecologists studying interspecific interactions or predator-prey dynamics. To be sure, morphological and isotopic diet analyses suffer from these same three drawbacks, but metabarcoding in particular has the potential to detect items in the scat that appear at microscopic levels. Thus, the rates of these errors are likely higher, though the magnitude is unknown. More research is needed on this topic for all of these diet analysis methods. A final drawback is the higher cost compared to other methods. Due to constraints on cost, we were not able to sequence every available scat, which limited our sample sizes. However, the costs of DNA metabarcoding have gone down precipitously over the last decade, and cost may not be a deterrent for much longer. Additionally, metabarcoding is less labor-intensive than morphological analysis, and may not be more expensive considering the entire workflow.

Due to the different advantages and disadvantages associated with the different methodologies, the best method to analyze the diet from scat samples will depend on the research questions. For example, morphological analysis detected most mammals to Species, and it detected mammals in some scats that metabarcoding did not detect. Studies interested only in detecting mammals in the diet may benefit from the morphological method. Conversely, the metabarcoding method should be used when more sensitivity is needed in identifying birds, plants, invertebrates, and anthropogenic food. Diet results also differed between molecular and morphological analysis of the same black bear samples (Bonin et al., 2020) and they recommended using both methods to gain a comprehensive understanding of omnivore diets.

## Conclusion

We found that the urban coyote diet is more diverse than the non-urban diet. Urban coyotes consumed a variety of naturalistic food items and there is no indication that they rely on anthropogenic food to meet their energy needs. More human food was found in the urban coyote diet, but anthropogenic food use was not significantly correlated with the proportion of impervious surface associated with each sample. Urban coyotes consumed deer, but it was much more prevalent in the non-urban coyote diet. As predicted, the metabarcoding diet analysis allowed us to detect more species than the morphological analysis. The metabarcoding approach enabled us to gain a more comprehensive understanding of the diet of an urban predator, and can be a useful tool for population management. Furthermore, this study underscores the importance of maintaining biodiversity within urban parks and other green spaces. Cities should also designate undeveloped green space, which is vital ecologically but often forgotten compared to maintaining developed (*i.e*., recreational) green space.

## Supplemental Information

10.7717/peerj.13788/supp-1 Supplemental Information 1 The percent impervious surface contained in 5 km and 10 km buffers around each sample. Samples were categorized as urban (red) if they contained 30-80% impervious surface and were categorized as non-urban (blue) if they contained 1–10% impervious surface within 5 km and 10 km buffers.

10.7717/peerj.13788/supp-2 Supplemental Information 2 The percentage of anthropogenic reads in each sample at varying levels of impervious surface in New York.

10.7717/peerj.13788/supp-3 Supplemental Information 3 Summary of the samples included in each sequencing run. NC = negative control.

10.7717/peerj.13788/supp-4 Supplemental Information 4 Fecal samples that were examined using both morphological analysis (Duncan et al., 2020) and metabarcoding (Henger et al. 2022).

10.7717/peerj.13788/supp-5 Supplemental Information 5 Human population density for all sample collection sites. Census data collected from https://www.census.gov/quickfacts/fact/table/US/PST045221.

## Additional Information and Declarations

### Field Study Permissions

The following information was supplied relating to field study approvals (*i.e*., approving body and any reference numbers): Sample collection at NYC parks approved by New York City Parks Department (NYS Permit #1118). No permit was necessary to sample at the non-urban sites because we were not collecting tissue or trapping animals.

### Data Availability

The following information was supplied regarding data availability: The raw data is available at GenBank: PRJNA792402. The code is available at GitHub: https://github.com/carolhenger/Coyote-Diet. https://www.ncbi.nlm.nih.gov/bioproject/PRJNA792402.
