# Supplementary material for: DNA metabarcoding reveals that coyotes in New York City consume wide variety of native prey species and human food
Source: PeerJ. 2022 Sep 21;10:e13788. doi: 10.7717/peerj.13788 (PMC9508883; doi:10.7717/peerj.13788)
Supplement: Supplemental Information 3 [file peerj-10-13788-s003.docx]

| Sample | Sequencing Run 1 Date of PCR | Sequencing Run 2 Date of PCR |
| --- | --- | --- |
| BP3 | NA | 4/22/18 |
| BP6 | 12/11/17 | 6/9/18 |
| BS1 | NA | 6/3/18 |
| CT1 | 1/10/18 | 6/9/18 |
| CT2 | 1/10/18 | NA |
| CT3 | 1/10/18 | 6/9/18 |
| CT4 | NA | 6/3/18 |
| CT5 | NA | 6/3/18 |
| EJ1 | 1/10/18 | NA |
| EJ2 | NA | 4/22/18 |
| EJ10 | 1/10/18 | 6/9/18 |
| EJ26 | NA | 6/6/18 |
| EJ28 | NA | 6/6/18 |
| EJ32 | NA | 6/6/18 |
| EJ34 | NA | 4/22/18 |
| EJ39 | NA | 6/6/18 |
| EJ40 | 1/10/18 | NA |
| EJ46 | 1/10/18 | 6/9/18 |
| EJ51 | 1/10/18 | 6/9/18 |
| EJ54 | 1/10/18 | 6/9/18 |
| FP4 | NA | 4/22/18 |
| FP5 | 1/10/18 | NA |
| FP7 | 1/10/18 | 6/9/18 |
| FP8 | 1/10/18 | 6/9/18 |
| FP9 | 1/10/18 | 6/9/18 |
| FP15 | NA | 4/22/18 |
| HS1 | 12/27/17 | 6/9/18 |
| HS2 | 1/10/18 | 6/9/18 |
| HS3 | 12/27/17 | 6/9/18 |
| HS4 | 12/27/17 | 6/9/18 |
| HS5 | NA | 6/3/18 |
| HS6 | NA | 6/3/18 |
| HS7 | NA | 6/3/18 |
| HS8 | NA | 6/3/18 |
| MRGP13 | 12/27/17 | 6/9/18 |
| MRGP221 | NA | 6/1/18 |
| MRGP174 | NA | 6/1/18 |
| MRGP239 | NA | 6/1/18 |
| MRGP241 | NA | 6/1/18 |
| MRGP251 | NA | 6/1/18 |
| MRGP350 | NA | 6/1/18 |
| MRGP395 | 12/27/17 | 6/9/18 |
| MRGP399 | 12/27/17 | 6/9/18 |
| MRGP401 | 12/27/17 | 6/9/18 |
| MRGP414 | 12/27/17 | 6/9/18 |
| MRGP428 | NA | 6/1/18 |
| MRGP570 | 1/10/18 | 6/9/18 |
| BX1 | 12/15/17 | 6/9/18 |
| BX2 | 12/15/17 | 6/9/18 |
| BX3 | 12/15/17 | 6/9/18 |
| BX4 | 12/15/17 | 6/9/18 |
| BX5 | 12/15/17 | 6/9/18 |
| BX6 | 12/15/17 | 6/9/18 |
| BX8 | NA | 6/6/18 |
| BX9 | NA | 6/6/18 |
| BX10 | NA | 6/6/18 |
| NC JAN 10 | 1/10/18 | NA |
| NC APR 22 | NA | 4/22/18 |
| NC JUN 1 | NA | 6/1/18 |
| NC JUN 3 | NA | 6/3/18 |
| NC JUN 6 | NA | 6/6/18 |
| NC JUN 9 | NA | 6/9/18 |
| NC DEC 4 | 12/4/17 | NA |
| NC DEC 11 | 12/11/17 | NA |
| NC DEC 15 | 12/15/17 | NA |
| NC DEC 27 | 12/27/17 | NA |
| NC DEC 29 | 12/29/17 | NA |
| PBP10 | 12/15/17 | 6/9/18 |
| PBP13 | 12/4/17 | 6/9/18 |
| PBP15 | NA | 4/22/18 |
| PBP19 | 12/4/17 | 4/22/18 |
| PBP23 | 12/4/17 | 6/9/18 |
| PBP27 | NA | 6/9/18 |
| PBP32 | NA | 6/9/18 |
| PBP33 | 12/11/17 | 6/9/18 |
| PBP41 | NA | 4/22/18 |
| PBP42 | NA | 4/22/18 |
| PBP43 | 12/4/17 | 6/9/18 |
| PBP44 | 12/4/17 | NA |
| PBP45 | 12/4/17 | NA |
| PC1 | 12/11/17 | 6/9/18 |
| PC2 | 12/15/17 | 6/9/18 |
| PC3 | 12/4/17 | 6/9/18 |
| PC5 | 12/15/17 | 6/9/18 |
| PC6 | 12/4/17 | 6/9/18 |
| PC7 | 12/11/17 | 6/9/18 |
| PC8 | NA | 4/22/18 |
| PC9 | 12/11/17 | 6/9/18 |
| PC10 | 12/11/17 | 6/9/18 |
| PC11 | 12/15/17 | 6/9/18 |
| PP1 | NA | 6/3/18 |
| PP9 | NA | 6/3/18 |
| RD2 | NA | 4/22/18 |
| RD4 | NA | 4/22/18 |
| RD5 | NA | 4/22/18 |
| RD6 | 1/10/18 | 6/9/18 |
| RD9 | 1/10/18 | 6/9/18 |
| RD10 | NA | 4/22/18 |
| RD11 | NA | 4/22/18 |
| RD12 | NA | 4/22/18 |
| RD14 | NA | 4/22/18 |
| RD15 | NA | 4/22/18 |
| RD18 | NA | 4/22/18 |
| RD23 | NA | 4/22/18 |
| RD25 | NA | 4/22/18 |
| RD26 | NA | 4/22/18 |
| RRP3 | NA | 6/6/18 |
| RRP4B | NA | 4/22/18 |
| RRP4 | NA | 4/22/18 |
| RRP5 | NA | 4/22/18 |
| RRP6 | NA | 4/22/18 |
| SR3 | 12/11/18 | 6/9/18 |
| SR9 | 12/11/18 | 6/9/18 |
| SR11 | NA | 6/6/18 |
| SR19 | NA | 6/6/18 |
| SR24 | NA | 6/6/18 |
| SR29 | NA | 6/6/18 |
| ST3 | NA | 6/3/18 |
| ST4 | NA | 6/3/18 |
| ST12 | NA | 6/3/18 |
| SV5 | NA | 6/3/18 |
| VC1 | 12/29/17 | 6/9/18 |
| VC3 | 12/29/17 | 6/9/18 |
| VC6 | 12/29/17 | 6/9/18 |
| VC7 | 12/29/17 | 6/9/18 |
| VC10 | 12/29/17 | 6/9/18 |
| VC12 | NA | 6/6/18 |
| VC17 | 12/29/17 | 6/9/18 |
| VC20 | 12/29/17 | 6/9/18 |
| VC23 | NA | 6/9/18 |
| VC24 | 12/29/17 | 6/9/18 |
| VC25 | 12/29/17 | 6/9/18 |
| VC26 | 12/29/17 | 6/9/18 |
| VC29 | 12/29/17 | 6/9/18 |
| VC34 | NA | 6/6/18 |
| VC38 | NA | 6/9/18 |
| WPR78 | 12/27/17 | NA |
