# Supplementary material for: DNA metabarcoding reveals that coyotes in New York City consume wide variety of native prey species and human food
Source: PeerJ. 2022 Sep 21;10:e13788. doi: 10.7717/peerj.13788 (PMC9508883; doi:10.7717/peerj.13788)
Supplement: Supplemental Information 4 [file peerj-10-13788-s004.docx]

| Sample | Duncan et al. 2020 | Henger et al. 2022 |
| --- | --- | --- |
| EJ10 | leaf litter, unknown mammal, rock | raccoon, plants, pig, fly |
| EJ26 | grass, leaf litter, unknown mammal | grape, cow |
| EJ28 | insect, leaf litter, rat, paper | plants |
| EJ32 | grass, unknown mammal | rabbit, bobcat, plants, pea, chicken |
| EJ34 | bird, insect, mollusk, rodent | plants |
| EJ39 | rat | rat, raccoon |
| EJ40 | insect | plants, grass, chicken |
| EJ46 | bird, leaf litter, unknown mammal, cloth | laughing gull, pigeon/dove, brown rat, raccoon, plants, grass, cow, soybean, chicken, fly |
| EJ51 | foil, fruit or seed, grass, insect | pigeon/dove, rat, opossum, chicken, pig, salamander |
| EJ54 | cloth/fiber, grass, insect, mammal | pigeon/dove, plant, cow, chicken, turkey, beetle, grass |
| FP4 | bird, plants, muskrat, plastic, rodent | plants, beetle |
| FP5 | bird, domestic cat, leaf litter, Microtus | opossum, skunk, cricket |
| FP7 | bird, grass | plant, chicken, grass |
| PC1 | muskrat, bird, leaf litter | dabbling ducks, starling, meadow vole, muskrat, deer, plants, chicken, salamander, grass |
| PC2 | grass, leaf litter, rabbit, chipmunk | rabbit, plants, rice, fly, grass |
| PC5 | bird, domestic cat, leaf litter | deer, plants, cat, chicken, pig, salamander, grass |
| PC6 | leaf litter, muskrat | white-footed mouse, meadow vole, muskrat, rat, plants |
| PC7 | muskrat | dabbling ducks, white-footed mouse, meadow vole, muskrat, plants, turkey, pig, fly |
| PC9 | leaf litter, muskrat | meadow vole, muskrat, plants, rice, grass |
| PC11 | bird, domestic cat | plant, soybean, domestic cat, chicken, fly, grass |
| NY1 | leaf litter, squirrel | plant, cow, chicken, turkey, pig, grass |
| NY2 | fruit/seed, insect, squirrel | squirrel, chipmunk, plant, chicken, fly, beetle |
| NY3 | grass, insect, raccoon | chicken, raccoon, plant, beetle, fly, grass |
| NY4 | fruit/seed, grass, leaf litter, plastic, raccoon | rabbit, raccoon, plant, chicken, beetle, grass |
| NY5 | fruit/seed, leaf litter | grass, soybean, chicken, pig, grass |
| NY6 | plastic, squirrel, rabbit | rabbit, plant, chicken, turkey, pig, fly |
| NY8 | bird, fruit/seed, insect | fly |
| NY9 | bird, fruit/seed, rubber, rabbit | plant, fly, cricket |
| BP3 | glass, leaf litter, meadow vole, white-tailed deer, raccoon | meadow vole, plants, grape |
| BP6 | leaf litter, deer | deer, plants, pig |
| PBP10 | fruit/seed, leaf litter, rabbit | plant, chicken |
| PBP13 | fruit/seed, leaf litter, muskrat | muskrat, meadow vole, raccoon, pig |
| PBP15 | bird, northern short tailed shrew, leaf litter, brown rat | Swainson's thrush, plants, domestic cat |
| PBP19 | fruit/seed, deer | rice, cat, turkey, pig, prawn |
| PBP23 | leaf litter, deer, squirrel | plants, deer, redear sunfish |
| PBP27 | bird, grass, insect, leaf litter, deer | chipmunk, deer, plants, chicken |
| PBP32 | deer, squirrel, rabbit | raccoon, plants, rice, beetle |
| PBP33 | fruit/seed, insect, leaf litter, deer | deer, chicken, fly |
| PBP41 | bird, fruit/seed, grass, meadow vole, rabbit | plants, beetle |
| PBP42 | bird, fruit/seed, grass, leaf litter, meadow vole, paper | meadow vole, plants |
| SR3 | leaf litter | dabbling ducks, starling, meadow vole, grass, chicken, pig, helmeted guineafowl, fly |
| SR9 | bird, leaf litter | dabbling duck, plant, goat, chicken |
| SR11 | deer | plant, grass, goat |
| SR19 | bird, grass, leaf litter, vole | plant, helmeted guineafowl, fly |
| SR24 | opossum, grass, leaf litter | plant, goat |
| SR29 | bird | plant, chicken, helmeted guineafowl |
| RD2 | leaf litter, raccoon | crow, pigeon/dove, raccoon, plant, chicken |
| RD4 | insect, leaf litter, raccoon | raccoon |
| RD5 | bird, raccoon | raccoon, plant |
| RD6 | bird, grass, raccoon | pigeon/dove, meadow vole, raccoon, chicken |
| RD9 | leaf litter, squirrel | pigeon/dove, plant, squirrel, chicken, beetle, moth/butterfly |
| RD18 | fruit/seed, grass, rabbit | raccoon, plants |
| RRP3 | bird, leaf litter, unknown mammal | plant, prunus, chicken |
| RRP4 | leaf litter, raccoon | thrush, raccoon, plant |
| RRP5 | leaf litter, squirrel | plant |
| RRP6 | bird, leaf litter, unknown mammal, paper | beetle |
| VC1 | leaf litter, squirrel, leather | squirrel, plant, chicken, fly, salamander |
| VC3 | bird, insect, leaf litter, squirrel | dabbling ducks, starling, turkey, rabbit, meadow vole, raccoon, plants, chicken, beetle |
| VC6 | seed, grass, insect, plastic, squirrel | raccoon, plant, chicken, fly |
| VC7 | grass, squirrel | raccoon, plant, fly, grass |
| VC10 | insect, leaf litter, squirrel | hawk, rat, raccoon, deer, plant, chicken, pig, fly |
| VC12 | leaf litter, raccoon | plant, prunus, chicken, pig |
| VC20 | bird, fruit/seed, leaf litter | plant, chicken, grass |
| VC23 | raccoon, cloth | deer, plant, chicken |
| VC24 | opossum | crow, raccoon, skunk, chicken |
| VC25 | rabbit | rabbit, plants, chicken, grass |
| VC26 | fruit/seed, leaf litter, rabbit | rabbit, plant, chicken |
| VC29 | bird, rabbit | rabbit, chicken, fly |
