# Supplementary material for: DNA metabarcoding reveals that coyotes in New York City consume wide variety of native prey species and human food
Source: PeerJ. 2022 Sep 21;10:e13788. doi: 10.7717/peerj.13788 (PMC9508883; doi:10.7717/peerj.13788)
Supplement: Supplemental Information 5 — Census data collected from https://www.census.gov/quickfacts/fact/table/US/PST045221. [file peerj-10-13788-s005.docx]

| Collection Site | Urban Categorization | County | State | Human Population/  Sq Mile |
| --- | --- | --- | --- | --- |
| Pelham Bay Park | Urban | Bronx | NY | 32,903.6 |
| Van Cortlandt Park | Urban | Bronx | NY | 32,903.6 |
| Bronx Park | Urban | Bronx | NY | 32,903.6 |
| Elmjack Ballfield | Urban | Queens | NY | 20,767.14 |
| Ferry Point Park | Urban | Bronx | NY | 32,903.6 |
| Soundview Park | Urban | Bronx | NY | 32,903.6 |
| Riverdale Park | Urban | Bronx | NY | 32,903.6 |
| Railroad Park | Urban | Queens | NY | 20,767.14 |
| Inwood Hill Park | Urban | New York | NY | 71,340.60 |
| Pugsley Creek Park | Urban | Bronx | NY | 32,903.6 |
| Stamford Residence | Non-urban | Fairfield | Connecticut | 1,467.2 |
| Palisade Park | Urban | Bergen | New Jersey | 3,884.5 |
| Butler Sanctuary | Non-urban | Westchester | NY | 2,204.7 |
| Ward Pound Ridge Reservation | Non-urban | Westchester | NY | 2,204.7 |
| Mianus River Gorge Preserve | Non-urban | Westchester | NY | 2,204.7 |
| Sterling Forest State Park | Non-urban | Orange | NY | 459.3 |
| Harriman State Park | Non-urban | Rockland | NY | 1,795.9 |
